# Impacts of late Quaternary environmental change on the long-tailed ground squirrel (*Urocitellus undulatus*) in Mongolia

**DOI:** 10.24272/j.issn.2095-8137.2018.042

**Published:** 2018-03-08

**Authors:** Bryan S. McLean, Batsaikhan Nyamsuren, Andrey Tchabovsky, Joseph A. Cook

**Affiliations:** 1 University of Florida, Florida Museum of Natural History, Gainesville, FL 32611, USA; 2 Department of Biology, School of Arts and Sciences, National University of Mongolia, Ulaan Baatar 11000, Mongolia; 3 Laboratory of Population Ecology, A.N. Severtsov Institute of Ecology and Evolution, Moscow 119071, Russia; 4 University of New Mexico, Department of Biology and Museum of Southwestern Biology, Albuquerque, NM 87131, USA

**Keywords:** Central Asia, Gobi Desert, Great Lakes Depression, Mongolia, Phylogeography

## Abstract

Impacts of Quaternary environmental changes on mammal faunas of central Asia remain poorly understood due to a lack of comprehensive phylogeographic sampling for most species. To help address this knowledge gap, we conducted the most extensive molecular analysis to date of the long-tailed ground squirrel (*Urocitellus undulatus* Pallas 1778) in Mongolia, a country that comprises the southern core of this species’ range. Drawing on material from recent collaborative field expeditions, we genotyped 128 individuals at two mitochondrial genes (cytochrome *b* and cytochrome oxidase I; 1 797 bp total). Phylogenetic inference supports the existence of two deeply divergent infraspecific lineages (corresponding to subspecies *U. u. undulatus* and *U. u. eversmanni*), a result in agreement with previous molecular investigations but discordant with patterns of range-wide craniometric and external phenotypic variation. In the widespread western *eversmanni* lineage, we recovered geographically-associated clades from the: (a) Khangai, (b) Mongolian Altai, and (c) Govi Altai mountain ranges. Phylogeographic structure in *U. u. eversmanni* is consistent with an isolation-by- distance model; however, genetic distances are significantly lower than among subspecies, and intra-clade relationships are largely unresolved. The latter patterns, as well as the relatively higher nucleotide polymorphism of populations from the Great Lakes Depression of northwestern Mongolia, suggest a history of range shifts into these lowland areas in response to Pleistocene glaciation and environmental change, followed by upslope movements and mitochondrial lineage sorting with Holocene aridification. Our study illuminates possible historical mechanisms responsible for *U. undulatus* genetic structure and contributes to a framework for ongoing exploration of mammalian response to past and present climate change in central Asia.

## INTRODUCTION

*Urocitellus undulatus* Pallas 1778 is a charismatic, medium-bodied ground-dwelling sciurid distributed across central Asia, including portions of Siberia, Mongolia, northwestern China, and easternmost Kazakhstan and Kyrgyzstan (Helgen et al., 2009; Kryštufek & Vohralík, 2013; Ognev, 1947; Wilson & Reeder, 2005). Although the genus *Urocitellus* (comprised of 12 species formerly subsumed within *Spermophilus*; Helgen et al., 2009) is distributed across much of the Holarctic region, *U. undulatus* is the only exclusively Palearctic species in this clade (Wilson & Reeder, 2005; McLean et al., 2016b). To date, various single- and multilocus investigations (Tsvirka et al., 2008; Ermakov et al., 2015; McLean et al., 2016b; Simonov et al., 2017) have revealed that *U. undulatus* is comprised of two deeply divergent lineages recognizable as well-defined subspecies (Kryštufek & Vohralík, 2013) or semi-species (Pavlinov & Lissovsky, 2012). These are an eastern lineage (*undulatus*) in western and central Siberia, northern Mongolia, and the Amur region of southeastern Siberia, and a western lineage (*eversmanni*) in western Mongolia, northern China, and Kazakhstan. However, our understanding of the full diversity and evolutionary and biogeographic history of this species across its vast range remains incomplete.

Although *U. undulatus* has been the subject of persistent morphological and molecular focus over the past three decades (Ermakov et al., 2015; Linetskaya & Linetskii, 1989; McLean et al., 2016b; Simonov et al., 2017; Tsvirka et al., 2008; Vorontsov et al., 1980), more expansive genetic datasets are necessary to test existing taxonomic hypotheses and illuminate the historical demography and biogeography of this species. Unfortunately, however, a lack of spatially comprehensive sampling and associated genetic data exists for this and many other central Asian vertebrate taxa. This data gap precludes identification of the broader abiotic and biotic processes acting to shape phylogeographic patterns and vertebrate community structure across this expansive region. For taxa with relatively high morphological conservatism (such as *Urocitellus*), such datasets are particularly crucial to refine our understanding of the true genomic and biogeographic histories of lineages.

The most significant lack of phylogeographic sampling from *U. undulatus* is in Mongolia, a country that nevertheless comprises the southern core of this species range. Several pressing evolutionary and biogeographical questions hinge on improved genetic sampling of Mongolian populations. First, although each of the subspecific lineages of *U. undulatus* (*undulatus* and *eversmanni*) is documented within the country, what are their exact geographic distributions? Second, do any populations in Mongolia display patterns of mixed mtDNA ancestry and, if so, where are these populations located? Third, how have known late Quaternary environmental changes shaped phylogeographic structure within the widespread western lineage (*eversmanni*)? Specifically, this lineage occupies an environmentally and climatically heterogeneous range in Mongolia, including multiple mountain systems (Khangai, Mongolian Altai, Govi Altai) that were subject to late Pleistocene glaciation, downward expansion of permafrost, and other environmental changes.

In this paper, we present the most comprehensive molecular phylogeographic analysis of *U. undulatus* in Mongolia to date. Drawing on material collected during expeditions of the Museum of Southwestern Biology (New Mexico, USA) from 1999–2016, we genotyped samples from across the entire Mongolian range of *U. undulatus* at two mitochondrial genes (cytochrome *b* (cyt *b*) and cytochrome oxidase I (*COI*), 1 797 bp total). We document population genetic variation and structure, and use those data to explore the potential effects of known late Pleistocene environmental changes on genetic patterns. Our work provides new information on the evolutionary and biogeographic history of *U. undulatus* in western Mongolia and lays a foundation for further analyses in this and similarly distributed central Asian mammals.

## MATERIALS AND METHODS

### Samples and sequencing

Specimens used in this study are housed at the University of New Mexico Museum of Southwestern Biology (MSB). Mongolian samples were collected during joint MSB-National University of Mongolia expeditions in 1999 (Tinnin et al., 2002), 2009–2012 (with University of Kansas and University of Nebraska), and 2015–2016 (with Northern Michigan University). Cumulative efforts of these expeditions include >6 500 cataloged mammal specimens from across major Mongolian vegetative and faunal provinces, many of which are associated with ecto- and endoparasite specimens archived at MSB Division of Parasites or University of Nebraska Manter Lab of Parasitology. All field methods followed guidelines of institutional animal care and use committees as well as the American Society of Mammalogists Guide for Use of Wild Mammals in Research (Sikes et al., 2011), and were focused on collection of “holistic” mammal specimens (e.g., Dunnum & Cook, 2012; McLean et al., 2016a; Yates et al., 1996). Cumulatively, these materials represent an unparalleled resource for establishing Mongolian faunal baselines in an era of ongoing global climate and environmental change.

**Figure 1:**
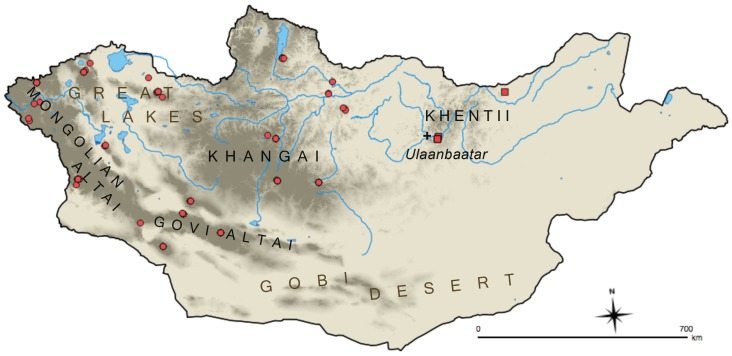
Map of Mongolia showing major landscape features and sampling localities of *U. undulatus*

We selected 128 specimens of *U. undulatus* for sequencing and analysis (Figure 1; Supplementary Table S1; GenBank accession Nos. MG883400–MG883654). The dataset included 119 individuals from 11 different Mongolian aimags (political land designations analogous to provinces or states) as well as putative representatives of both subspecies (as delineated by Kryštufek & Vohralík, 2013). The dataset also included nine individuals of *U. u. undulatus* from Sakha Republic in northern Siberia. We selected sequences of the Columbian ground squirrel (*U. columbianus*) from GenBank as the outgroup for phylogenetic analysis. Frozen tissue samples of all *U. undulatus* individuals (liver, muscle, or dried muscle) were subjected to lysis in a solution of 600 μL tissue lysis buffer and 12–15 μL reconstituted proteinase K (Omega E.Z.N.A. kit; Omega Bio-tek, Inc., USA) for up to 24 hours. Genomic DNA was isolated using a standard salt/ethanol extraction procedure. To reduce the potential for PCR inhibition, all dried muscle samples were processed prior to lysis by removing debris, cutting into sub-centimeter sized pieces, and washing in 100% ethanol for 15 min at room temperature, vortexing several times; these were then washed in STE buffer under refrigeration for 12–16 h. Final extractions were quantified flourometrically using a Qubit Broad Range assay kit (Life Technologies Corp., USA).

Higher elevations are shown in darker colors and major mountain ranges and landscape features are indicated with text. Collection localities of samples used in this study indicated by red circles (*U. u. eversmanni*) and squares (*U. u. undulatus*).

We used the primer pair MVZ05/MVZ14 (5′-CGAAGCTTGATATGAAAAACCATCGTTG/CTTGATATGAAAAACCATCGTTG-3′; Smith & Patton, 1993) to amplify all 1 140 bp of the mitochondrial cyt *b* gene. We used the primer pair HCO2198/LCO1490 (5′-TAAACTTCAGGGTGACCAAAAAATCA/GGTCAACAAATCATAAAGATATTGG-3′; Folmer et al., 1994) to amplify 657 bp of the mitochondrial *COI* gene. Amplification of both mtDNA regions took place in 25 μL reactions, with annealing temperatures of 52 °C and 48 °C, respectively. Purified PCR products were sequenced using Big Dye Terminator 3.1 technology (Applied Biosystems, USA) on an ABI 3130 automated DNA sequencer in the Molecular Biology Facility in the Department of Biology at University of New Mexico. Sequences were manually edited in Sequencher v5.3 (Gene Codes Corp., Michigan, USA) and aligned with MUSCLE v3.7 (Edgar, 2004) using default settings on the CIPRES science gateway (www.phylo.org; Miller et al., 2010).

We used the R packages pegas (Paradis et al., 2017b) and popGenome (Pfeifer et al., 2017) to calculate standard population genetic statistics, and to test for signals of population expansion based on the Tajima’s *D* statistic. Partial deletion of positions with missing data was performed when calculating pairwise nucleotide-based metrics (nucleotide diversity and pairwise number of nucleotide differences). We inferred phylogeny for all samples in a Bayesian framework. First, we used PartitionFinder v2.1 (Lanfear et al., 2017) to simultaneously infer the best-fit partitioning scheme and models of sequence evolution for the concatenated mtDNA matrix, as evaluated using the AICc metric. We conducted Bayesian phylogenetic inference in MrBayes v3.2.3 (Ronquist & Huelsenbeck, 2003) on CIPRES, using the optimal partitioning scheme inferred above. Two independent MCMC analyses composed of 4 Metropolis-coupled chains each (the default) were used to estimate posterior distributions of tree topologies, running both analyses for 10 000 000 generations, sampling every 1 000 generations, and discarding the first 25% of samples as burn-in. Convergence of all parameters was assessed in Tracer v1.6.0 (Rambaut et al., 2014) by visualizing trace plots and ensuring effective sample sizes >200.

To characterize population structure in *U. undulatus*, we performed an analysis of molecular variance (AMOVA) of all samples in the R package poppr (Kamvar et al., 2014). For each gene, we included subspecies (*undulatus*, *eversmanni*) and aimag of origin (12 total in our dataset) as factors. We also tested significance of the observed variance patterns with 4 999 randomizations. Because political boundaries may only weakly capture landscape-scale genetic structure, we next tested consistency of our data with an isolation-by-distance (IBD) model, focusing only on *U. u. eversmanni*. For that test, we used functions in the R packages ape (Paradis et al., 2017a) and raster (Hijmans et al., 2017) to compute pairwise genetic (*P*-values) and geographic (in meters) distances, respectively. We calculated correlations between these matrices using the mantel function in vegan (Oksanen et al., 2017) and assessed statistical significance using 4 999 permutations of the geographic distance matrix. Finally, we visualized spatial patterns of mtDNA diversity by computing a minimum-spanning haplotype network (Bandelt et al., 1999) in PopART (http://popart.otago.ac.nz), using just the significantly more variable cyt *b* gene.

## RESULTS

**Figure 2:**
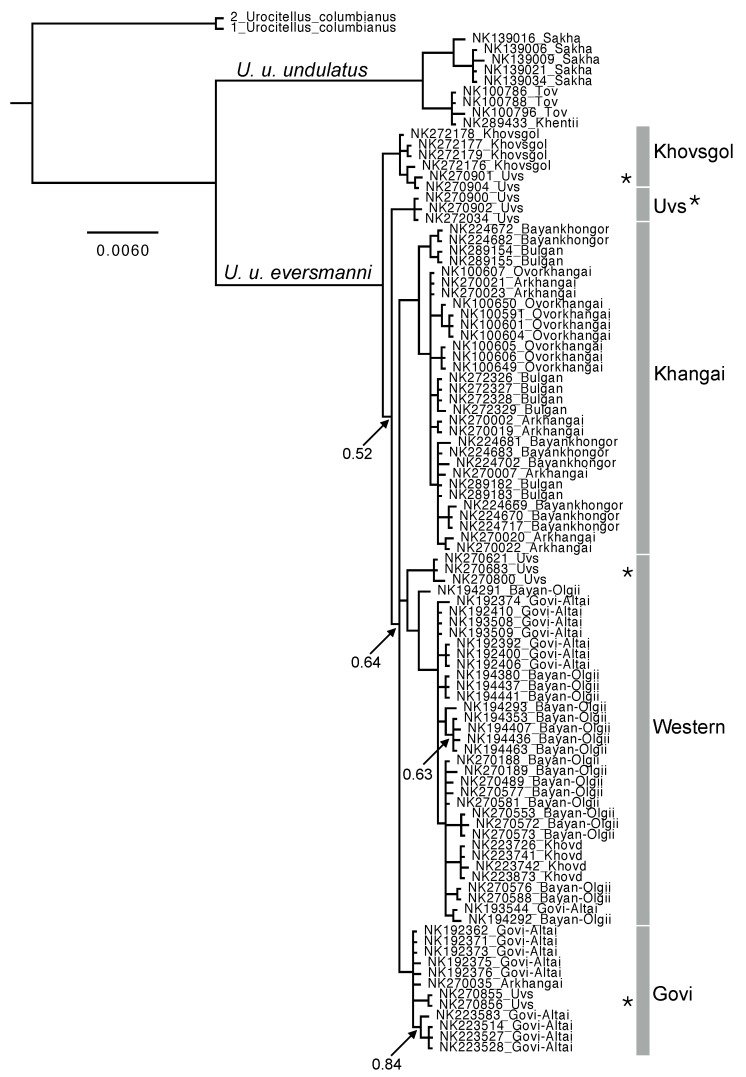
Majority-rule consensus phylogram of *Urocitellus undulatus* based on Bayesian inference in MrBayes

The best-fit partitioning scheme for the concatenated mtDNA matrix included a different partition for each codon position within the cyt *b* and *COI* genes (although codon position 2 for both genes shared the same partition; Table 1). The Tamura-Nei (TrN) substitution model or one of its extensions (TrN with equal base frequencies, gamma-distributed heterogeneity, and/or invariant sites) was preferred for all partitions (Table 1). Phylogenetic inference in MrBayes recovered two major clades (*U. u. undulatus* and *U. u. eversmanni* sensu Kryštufek & Vohralík, 2013) with strong support (PP=1; Figure 2). The average uncorrected genetic distance (mean±*SD*) between these clades is 5.84%±0.19 for cyt *b*, but a more modest 2.67%±0.20 for *COI*. Notably, the maximum inter-clade distance for *COI* (3.16%) is concordant with Ermakov et al.’s (2015) estimate of 3.5% using this same marker but different individuals. For reference, average uncorrected distances between all samples of *U. undulatus* and the two *U. columbianus* outgroups are 8.21%±0.14 and 4.99%±0.16 for cyt *b* and *COI*, respectively. However, we note that *U. undulatus* and *U. columbianus* may not share a most recent common ancestor (McLean et al., 2016b).

**Table 1:** Best-fit models of evolution according to the AICc metric for partitions of the concatenated mtDNA matrix

	Model	No. sites
cyt *b* position 1	TrNEF + G	380
cyt *b* position 2 + *COI* position 2	Trn + I	599
cyt *b* position 3	TrN + G	380
*COI* position 1	TrN	219
*COI* position 3	TrN	219

Subspecies and major clades (within *U. u. eversmanni* only) are indicated by text. The tree is rooted on the split from the Nearctic *U. columbianus*. Branches with posterior probabilities less than 0.9 (i.e., between 0.5 and 0.9) are indicated by arrows. Identical cyt *b* haplotypes are represented by a maximum of 3 individuals in the tree; additional duplicate haplotypes were trimmed for clarity (*n*=32). Asterisks denote individuals from Uvs Aimag.

Both the genotyping results (Table 2) and phylogenetic inference confirmed hypotheses that the *U. u. eversmanni* lineage is widespread across western Mongolia, occurring in far northern (Khövsgöl), southern (Govi Altai), and westernmost (Bayan-Ölgii) aimags. Conversely, the nominal eastern lineage (*U. u. undulatus*) occupies a more restricted range in the country; individuals taken from as far east as Khövsgöl Aimag, Bulgan Aimag and the southeastern Khangai Mountains maintain evolutionary affinity with the western *U. u. eversmanni* lineage (Figure 2). We found no evidence for mtDNA admixture (i.e., presence of haplotypes from multiple subspecies) in any population surveyed, including those proximate to the apparent phylogeographic break between *undulatus* and *eversmanni*, suggesting a persistent lack of gene flow between these two subspecific lineages.

**Table 2:** Population genetic summary statistics for *Urocitellus undulatus eversmanni*, partitioned by gene

*n*	*H*	*Hd*	*S*	*k*	π
cyt *b* (1 140 bp)					
110	47	0.91	67	9.60	8.71 × 10^−3^
*COI* (657 bp)					
111	22	0.88	23	2.16	3.29 × 10^−3^

*n*: number of samples, *H*: number of haplotypes, *Hd*: haplotype diversity, *S*: number of polymorphic sites, *k*: average number of nucleotide differences, π: nucleotide diversity.

Within the widespread *U. u. eversmanni* lineage, three geographically-associated subclades are strongly supported in the MrBayes topology, from (1) the Khangai Mountains and surrounding regions (“Khangai” clade); (2) the Mongolian and Govi Altai and surrounding highland regions (“Western” clade); and (3) additional ranges of the Govi Altai (“Govi” clade). Additional, more narrowly distributed genetic clusters were also recovered and include populations from Khövsgöl and Uvs Aimags (Figure 2), although we note that some clades exhibit incomplete lineage sorting. For example, individuals from Uvs Aimag are associated with four distinct genetic clusters or subclades in the Bayesian phylogeny (asterisks in Figure 2). Finer-scale associations with landscape features such as rivers were not evident in our dataset.

Despite the incomplete geographic sorting of mtDNA haplotypes and general lack of fine-scale population patterning, there is detectable phylogeographic structure within Mongolian *U. u. eversmanni*. AMOVAs support a role for broad provincial classifications in mtDNA variation, with 21.7% and 33.9% of molecular variance in cyt *b* and *COI*, respectively, attributable to aimag of origin after accounting for subspecific variation (Table 3). Both values are greater than expected by chance (Table 3). A statistically significant correlation was also found between matrices describing raw genetic distances and raw geographic distances within *U. u. eversmanni* in cyt *b* (r=0.58, *P*<0.01) and *COI* (r^2^=0.38, *P*<0.01), thereby supporting an isolation-by-distance hypothesis in this subspecies.

**Table 3:** Results of analysis of molecular variance (AMOVA) for both genes and all samples of *Urocitellus undulatus*

	Degrees of freedom	Sum of squared deviations	Variance	Variance relative to expected	*P*
	cyt *b*				
Between subspecies	1	2.84	0.05 (8.9)	greater	0.01
Among aimags	10	15.66	0.12 (21.7)	greater	0.01
Within aimags	115	42.88	0.37 (69.3)	less	0.01
Total	126	61.38	0.54 (100)		
	*COI*				
Between subspecies	1	4.25	0.08 (14.9)	greater	0.01
Among aimags	10	21.33	0.18 (33.9)	greater	<0.01
Within aimags	116	31.65	0.27 (51.2)	less	<0.01
Total	127	57.23	0.53 (100)		

**Figure 3:**
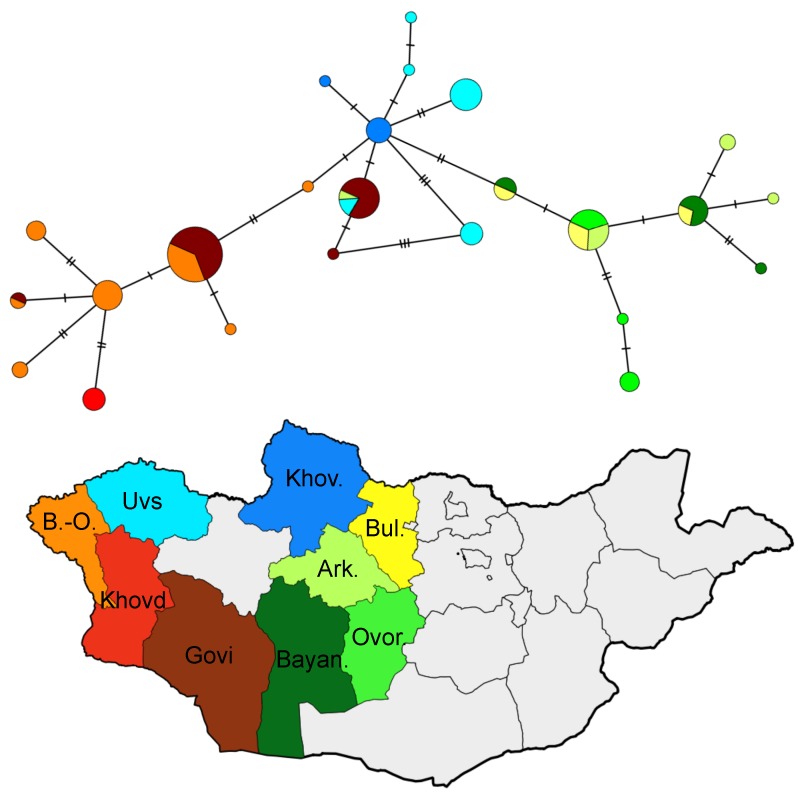
Minimum-spanning haplotype network of all *Urocitellus undulatus eversmanni* samples used in this study

Nevertheless, we emphasize that divergences among *U. undulatus* subclades are very low. This can be visualized in the minimum-spanning haplotype network (Figure 3), and is borne out in uncorrected pairwise genetic distances computed within the clade of (mean±*SD*) 0.63%±0.39 for cyt *b* and 0.33%±0.22 for *COI*. Those distances are roughly an order of magnitude lower than between *U. u. eversmanni* and *U. u. undulatus* (Ermakov et al., 2015; this study). They are also lower than those found within *U. u. eversmanni* populations from the adjacent Altai region of southern Russia (Simonov et al., 2017), although the latter study used the noncoding and more variable mtDNA control region. In addition, although there was more variation within than among aimags, AMOVAs suggest that there is a significantly lower amount of molecular variance within aimags than expected by chance, highlighting the shallow differentiation that exists in broad geographic regions. Finally, we recovered negative values of Tajima’s *D* for both genes in *U. u. eversmanni* (cyt *b*, *D*=–2.09; *COI*, *D*=–1.47) and across all samples of *U. undulatus* (cyt *b*, *D*=–1.52; *COI*, *D*=–0.82), although the result was only significant at the *P*<0.01 level for *U. u. eversmanni* cyt *b* (*P*>0.10 for all others).

Individuals in haplotype network (top) are colored by aimag of origin, with colors corresponding to the map (below). Notches indicate single nucleotide substitutions. Note the map does not include the distribution of *U. u. undulatus* (B.-O.=Bayan-Olgii, Govi=Govi-Altai, Khov.=Khovsgol, Bul.=Bulgan, Ark.=Arkhangai, Ovor.=Ovorkhangai, Bayan.=Bayankhongor).

## DISCUSSION

Relatively little is known about range-wide patterns of genetic structure and endemicity in many central Asian mammal species. This information gap precludes tests of existing taxonomic hypotheses and limits deeper knowledge of how past environmental changes across this vast region have influenced mammalian diversification. This is particularly true for ecomorphologically conservative taxa, such as *U. undulatus*, where molecules and morphology might be expected to give cryptic or conflicting historical signals. Indeed, the few phylogeographic investigations of non-volant vertebrate taxa (e.g., *Phrynocephalus* lizards, Wang & Fu, 2004; *Rhombomys* gerbils, Ning et al., 2007; *Bufo* toads, Zhang et al., 2008; *Meriones* gerbils and *Allactaga*, *Dipus* jerboas, Liao et al., 2016) in central Asia to date hint at significant impacts of late Pleistocene environmental change on population genetic diversity and geographic structure in this region. Our analysis of *U. undulatus* allows us to establish preliminary hypotheses of mammalian spatiotemporal response to late Quaternary change within Mongolia that can be further tested in this and other sympatric species.

On a range-wide scale, our results support the existing systematic hypothesis (Kryštufek & Vohralík, 2013; Pavlinov & Lissovsky, 2012) that *U. undulatus* is comprised of two deeply divergent lineages (*undulatus* and *eversmanni*). The strong phylogeographic break between these lineages, which stretches from southern Lake Baikal (Russia) through eastern Selenge and Töv Aimags (Mongolia), contrasts, however, with previously published patterns of cranial shape variation. Specifically, morphological studies (Kryštufek & Vohralík, 2013; Linetskaya & Linetskii, 1989; Vorontsov et al., 1980) found populations from Yakutia and the Amur region of Russia to be highly divergent in cranial shape, while populations from the remainder of the range extending across southern Siberia and northern Mongolia exhibited a broad longitudinal cline in cranial shape. Because body size varies significantly in *U. undulatus*, and cranial morphology is highly allometric in *Urocitellus* ground squirrels (e.g., Robinson & Hoffmann, 1975), it seems likely that cranial shape data (especially those based on linear measurements) largely reflect body size differences, which may or may not be useful for elucidating evolutionary structure in this species. More robust tests of species limits and phylogeographic hypotheses in *U. undulatus*, as well as of our assertion of a lack of gene flow between *undulatus* and *eversmanni*, await data from additional and independent regions of the nuclear genome.

Considering the *eversmanni* lineage which makes up the bulk of our sampling, our results provide new insights into effects of late Quaternary climate change on historical biogeography of this taxon across Mongolia. The record of late Pleistocene and Holocene environmental change in this region includes extensive plateau and mountain valley glaciation, specifically in the Khangai, Mongolian Altai, and Govi Altai ranges; extensive downward expansion of permafrost; and intermittent formation and draining of lakes at both higher and lower elevations (Böhner & Lehmkuhl, 2005; Grunert et al., 2000; Lehmkuhl, 1998; Lehmkuhl & Lang, 2001). Montane glaciation and the expansion of permafrost should have driven downslope range shifts in *U. undulatus*, as this species prefers mesic steppe habitats and requires deeper permafrost levels for construction of hibernacula. These range shifts, in turn, should have promoted increased mixing of populations across lowlands of western Mongolia between 30–12 kya.

Consistent with that scenario, we found shallow divergence between major mtDNA clusters within *U. u. eversmanni*. However, because many of these mitochondrial lineages are restricted to mid- and high-elevation steppe habitats (e.g., in the Govi Altai) and unlikely to experience high levels of gene flow, low divergences are likely to be signatures of past (i.e., latest Quaternary) population mixing across lowlands of western Mongolia. The shallow but significant geographic structure that does exist among geographically and ecologically disparate populations of *U. u. eversmanni* could, in turn, have been generated by partial lineage sorting and haplotypic divergence following expansion into more favorable areas and cessation of population connectivity.

Zhang et al. (2008) found a similar pattern of reduced haplotype and nucleotide diversity in green toads (*Bufo viridis*) inhabiting eastern Central Asia. Their data support a history of refugial isolation in montane regions of northwest China and eastern Kazakhstan followed by rapid postglacial expansion into surrounding basins. Liao et al. (2016) described a similar pattern in the jerboa *Allactaga sibirica* in China. Conversely, our data, including low population structure and negative values for Tajima’s *D* in *U. u. eversmanni*, suggest recent upslope range expansions from lowland refugia. Therefore, from an elevational perspective, these studies support opposite historical scenarios that likely reflect differences in the need of amphibians to track water availability versus that of steppe mammals. Gür (2013) and Liao et al. (2016) describe a third pattern in Anatolian ground squirrels (*Spermophilus xanthoprymnus*) in Turkey and gerbils and jerboas (*Meriones meridianus* and *Dipus sagitta*) in northern China, suggesting that these species expanded their areal, but not elevational, distributions during the late Pleistocene in conjunction with expansion of cold steppe habitats and deserts.

If our hypothesis of lowland Pleistocene range shifts is correct, the most extensive corridors for gene flow among ancient populations of *U. u. eversmanni* may have been in the “Great Lakes Depression” and “Valley of the Govi Lakes”. Those lowland regions, located between major Mongolian mountain ranges, are mostly contiguous with a broad longitudinal band of mesic steppe that transverses Mongolia and forms a corridor for more arid-adapted taxa such as Mongolian gazelle (*Procapra gutturosa*) and Tolai hare (*Lepus tolai*; Batsaikhan et al., 2014). However, Pleistocene environments in this region were likely more mesic than today and may have included a mixture of steppe and forest elements (Grunert et al., 2000; Böhner & Lehmkuhl, 2005). Downward expansion of mesic floral and faunal elements into the Great Lakes Depression during the late Pleistocene would have provided suitable habitat for an increasingly mesic-adapted suite of vertebrate species such as *U. undulatus*.

As a post hoc investigation of this scenario, and to more thoroughly parse AMOVA results, we calculated population genetic statistics (haplotype and nucleotide diversity) for all aimags with at least 15 sampled individuals (Uvs, Bayan-Ölgii, and Govi Altai). While Uvs Aimag contains lower haplotype diversity (0.71) than either Bayan-Ölgii or Govi Altai aimags (0.91 and 0.80, respectively), it has higher nucleotide diversity (6.69×10^−3^ vs. 2.37×10^−3^ and 4.92×10^−3^, respectively). This increased nucleotide polymorphism could have resulted from confinement of multiple *U. u. eversmanni* lineages within a Pleistocene refugium spread across the Great Lakes Depression and surrounding basins, followed by rapid range expansion into favorable montane habitats that became increasingly disjunct with Holocene climate changes, yielding the phylogeographic and demographic signals we detected. Simonov et al. (2017) demonstrated elevated mtDNA diversity in *U. u. eversmanni* from the southern Altai Mountains in Russia, and suggested that those populations may also have been isolated in lowland glacial refugia. Our data strongly support their hypothesis that one of these refugia was in the Great Lakes Depression. However, we cannot completely rule out refugia elsewhere in northern Mongolia, such as in Khövsgöl Aimag, a region proximate to the Great Lakes Depression, but from which we were only able to sample six individuals from a relatively small area.

Currently, Pleistocene paleoenvironments of western Mongolia are somewhat poorly constrained, preventing more precise and detailed links between small mammal historical biogeography and past environmental change. Grunert et al. (2000) proposed a lowstand (i.e., lowered lake levels due to local or regional environmental change) for both Uvs Nuur and Bayan Nuur during the Last Glacial Maximum (LGM), which would have led to exposure of even more extensive areas in the Great Lakes Depression than are available today. While these lake lowstands are somewhat counterintuitive given the relatively mesic conditions inferred for other mid-latitude regions during the LGM, a similar pattern of glacial lowstands has been described from lakes in nearby northwestern China (Fang, 1991). Conversely, the southern Altai Mountains in Russia experienced formation of large glacial lakes during the late Pleistocene (e.g., Rudoy, 2002). Understanding the extent to which these idiosyncratic landscape-level responses interacted with regional-scale environmental variability to impact the distribution and demography of *U. undulatus* will require linking all currently available sequence datasets with new genetic data in a range-wide phylogeographic framework.

## CONCLUSION

We analyzed the phylogeography of the long tailed ground squirrel (*Urocitellus undulatus*) across the southern core of its large central Asian range. Phylogenetic and population genetic inferences based on mtDNA strongly support the presence of two major lineages in Mongolia (*U. u. undulatus* and *U. u. eversmanni*). Within the more widespread *U. u. eversmanni*, we identified statistically significant but extremely shallow phylogeographic structure, with modern genetic clusters associated with Mongolian mountain systems (Khangai Mountains, Mongolian and Govi Altai). Together, our analyses support a late Pleistocene history of extensive population admixture in *U. u. eversmanni*, possibly across the Great Lakes Depression and contiguous lowlands of north-west Mongolia, followed by geologically recent diversification in postglacial isolation. In addition to providing new geographic context for *U. undulatus* systematics and phylogeography, our study establishes hypotheses of distributional and demographic response to past environmental change in mesic-adapted central Asian mammal species which may be tested using robust, genomic-scale datasets.

## Supplementary Material

http://www.zoores.ac.cn/fileup/2095-8137/SUPPL/20180720193101.zip
